# Polyethylenimine/Graphene Oxide Nanocomposite for Lightweight X-Ray Radiation Shielding in Aerospace Applications

**DOI:** 10.3390/ma19173762

**Published:** 2026-09-04

**Authors:** Sabina Botti, Francesca Bonfigli, Flaminia Rondino, Dariush Hampai, Yury Cherepennikov, Sultan Dabagov

**Affiliations:** 1 Photonics Micro- and Nano-structures Laboratory, Physical Technologies and Security Division, Nuclear Department, ENEA, Via E. Fermi 45, 00044 Frascati, Italy; francesca.bonfigli@enea.it (F.B.); flaminia.rondino@enea.it (F.R.); 2 X-Lab, INFN–LNF, Via E. Fermi, 54, 00044 Frascati, Italy; dariush.hampai@lnf.infn.it (D.H.); yury.cherepennikov@lnf.infn.it (Y.C.); sultan.dabagov@lnf.infn.it (S.D.)

**Keywords:** graphene oxide, polyethylenimine, X-ray radiation shielding, Raman spectral imaging, Monte Carlo simulations

## Abstract

The demand for lightweight, flexible, and lead-free radiation shielding for next-generation extravehicular activity suits and aerospace habitat liners is rapidly intensifying. This study investigates the structural organization and X-ray attenuation performance (10–60 keV) of polyethylenimine/graphene oxide (PEI/GO) composites with GO loadings up to 50 wt%. Microstructural evolution was systematically tracked via optical image quantification (dispersion, connectivity, and local mixing indices) correlated with micro-Raman spectral mapping. Raman analysis confirmed a structural transition at 40 wt% GO, driven by a dynamic competition between covalent amine–epoxide/carboxyl functionalization and localized π–π stacking of sp^2^ domains. X-ray transmission and linear attenuation coefficients were evaluated using a dual approach, coupling experimental X-ray exposures with deterministic NIST XCOM calculations and stochastic Monte Carlo simulations. The results demonstrate that GO significantly amplifies low-energy photoelectric absorption due to its oxygen-rich functionalities. An anomalous thickness-dependent attenuation paradox, which can be explained by accounting for forward-scattered Compton buildup in thick blocks and spatial edge refraction along non-percolating cluster interfaces in thin films, was observed experimentally. These findings provide critical material design rules for advanced, flexible, and wearable photon shields operating without mass penalties.

## 1. Introduction

The increasing duration of crewed space missions and the emergence of long-term extraterrestrial habitats have intensified the demand for lightweight multifunctional materials capable of simultaneously providing structural performance, environmental resistance, and radiation protection. In low Earth orbit and deep-space environments, astronauts and sensitive payloads are exposed to complex radiation fields composed of galactic cosmic rays, solar particle events, secondary bremsstrahlung photons, and trapped energetic particles. The deployment of lightweight, non-toxic, and mechanically resilient materials for radiation protection is a critical priority in space exploration and modern aerospace engineering. Traditional shielding relies heavily on high-atomic-number metals, such as lead and aluminum, which pose significant drawbacks, including toxic environmental footprints, heavy weight penalties, and the generation of dangerous secondary bremsstrahlung radiation under high-energy electron bombardment.

To overcome these limitations, hydrogen-rich polymers have attracted considerable attention as candidate radiation shielding materials due to their effectiveness in attenuating charged particles and mitigating secondary neutron generation compared to the results for high-Z metallic systems. Among them, PEI has emerged as a promising engineering matrix because of its high nitrogen content, processability, thermal stability, adhesion to fibrous substrates, and compatibility with multifunctional nanofillers. In parallel, graphene-based materials, particularly GO, have shown significant potential for aerospace multifunctional systems owing to their exceptional mechanical reinforcement, tunable electronic properties, low density, thermal conductivity, and barrier behavior [1]. Compared to pristine graphene, GO is particularly attractive due to its oxygen-containing functional groups that enable strong interfacial interactions with polymer matrices and textile fibers, facilitating homogeneous dispersion and robust coating architectures [2]. Beyond mechanical reinforcement, GO has been increasingly investigated for electromagnetic interference shielding, thermal control coatings, atomic oxygen resistance, and radiation attenuation, making it an appealing platform for multifunctional coatings for aerospace applications.

Recent studies suggest that nanostructured carbon materials may contribute to photon attenuation through several mechanisms. In the low-energy X-ray regime, attenuation arises predominantly through photoelectric absorption and incoherent scattering, governed by electron density and effective atomic composition. In GO-based systems, oxygen functionalities contribute to increased local electron density and interfacial heterogeneity, which may enhance photon interaction probability compared to that of pristine graphene-based composites. At the same time, layered GO architectures can promote multiple scattering pathways and tortuous transport effects, potentially amplifying attenuation in thin-film geometries [3,4]. Despite their potential, the key physical mechanisms governing X-ray attenuation in light-element nanocomposites across different energy regimes remain insufficiently explored. Specifically, it is necessary to determine how filler loading, local oxygen-containing functional groups, and structural stacking influence photon interaction probability. Moreover, few studies have addressed the comparison between GO and pristine graphene within identical polymer platforms, especially under photon energies relevant to medical diagnostics, secondary space radiation, and X-ray exposure. Therefore, the interplay between nanofiller chemistry, composite morphology, and shielding efficiency warrants deeper investigation.

In this work, we investigated the X-ray attenuation efficiency, structural composition, and local structural ordering of PEI/GO composite across a broad range of GO filler concentrations. The evolution of the PEI/GO composite structure with GO content was investigated by combining Raman spectroscopy with quantitative image analysis, enabling a direct correlation between vibrational signatures and mesoscale organization. We demonstrated that amine-mediated chemical reduction occurring during composite preparation plays a key role in restoring local sp^2^ domains at high filler loadings. By using X-ray transmission radiography and XCOM Monte Carlo simulations, we evaluated the material’s linear attenuation coefficient. Our aim was to gain a deeper understanding of how the PEI/GO structure affects X-ray radiation shielding, combining experimental characterization with Monte Carlo modeling and XCOM-based theoretical analysis, contributing to the development of a new class of flexible, lightweight, lead-free, and multifunctional radiation shielding materials, bridging nanocomposite science and aerospace materials engineering.

## 2. Materials and Methods

### 2.1. Sample Preparation

Two-dimensional GO/PEI composites were prepared via a facile two-step fabrication process. GO (Merck (Darmstadt, Germany), 2 mg mL^−1^) was gradually added to an aqueous solution of branched PEI (PEI, average MW 25,000, Merck, 0.16 g/L) under vigorous stirring. Different GO loading was employed to obtain the desired GO weight percentage in the final composite, ranging from 1 to 50 wt%.

The resulting GO/PEI dispersion was then heated at 90 °C for 4 h to ensure a complete homogenization and uniform distribution of the GO suspension in the polymer.

**Figure 1:**
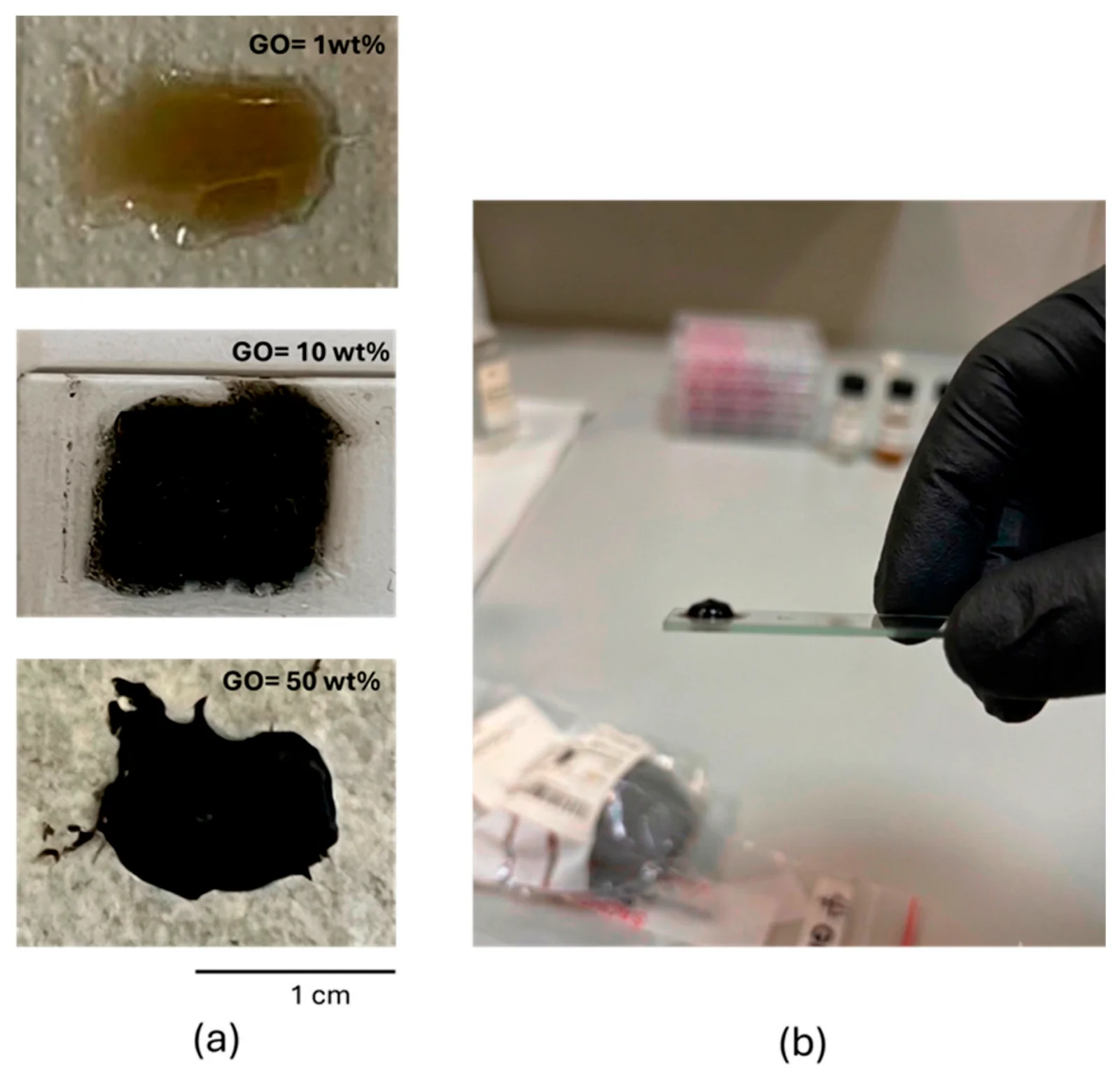
(**a**) Photographic images of PEI/GO membrane with GO contents of 1 wt%, 10 wt%, and 50 wt%; (**b**) photographic image of a freestanding PEI/GO tile-shaped structure with a GO content of 50 wt%.

Indeed, the thermal treatment guarantees the formation of effective interactions between the amine groups of PEI and the oxygen-containing functional groups on the GO sheets, consequently improving the homogeneity and the flexibility of the resulting composite [5,6]. After thermal treatment, the composite was deposited onto the desired support and dried under ambient conditions for 24 h. Upon drying, a freestanding PEI/GO membrane was obtained, namely a self-supporting film that can be handled while maintaining its structural integrity. Following the same procedure, samples with a high GO loading were successfully prepared in the form of a flexible tile-shaped structure. This result highlights the versatility of the PEI/GO composite and suggests its potential applicability across a broad range of applications requiring materials with variable shapes and mechanical properties (see Figure 1). No annealing step was performed after membrane formation.

### 2.2. Evaluation of Dispersion and Connectivity of the Composite Through Optical Images Analysis

To quantify the spatial organization of GO flakes within the polymer matrix, we analyzed the white light optical images using two complementary approaches in order to evaluate the dispersion and the connectivity index [7,8,9].

The dispersion index was evaluated following two different approaches [7,8]. In the first approach, each micrograph, in which GO domains appear as dark regions and the polymer matrix as brighter regions, was converted into an 8-bit grayscale image and normalized to remove contrast variations. A global thresholding procedure was then applied to generate binary images, in which the filler phase (GO) was identified as black pixels (value = 1), while the polymer matrix corresponded to white pixels (value = 0).

To quantify the flake dispersion, each binary image was subdivided into a grid of square subdomains of equal size. The local filler area fraction ϕ*_i_* was calculated for each subdomain as the ratio of filler pixels to the total number of pixels within the subdomain. The global average filler area fraction ϕ_avg_ with its standard deviation σ were then determined. The dispersion index D is calculate as follows:

(1) $$D=1-\;\frac{\sigma }{\emptyset_{avg}}$$

This parameter provides a measure of spatial homogeneity, i.e., for D→1, the uniformity of GO dispersion increases, while larger values indicate heterogeneity due to filler clustering or aggregation.

In the second approach, a grayscale-based algorithm was implemented to evaluate dispersion without binarization. For each pixel, the number of neighboring pixels with similar intensity (N_same_) and different intensity (N_diff_) was calculated. Pixels were considered similar when the intensity difference was below a specified threshold (ΔI < 0.05). Following this method, the dispersion parameter R was defined as

(2) $$R=\frac{N_{diff}}{N_{same}}$$

The final value of the dispersion index was then obtained by normalizing R with respect to the value corresponding to an ideally dispersed system. Unlike the first approach, this method captures local homogeneity and interfacial mixing, as high local contrast can arise both from well-dispersed fillers and from large, aggregated domains. Therefore, this parameter describes the dispersion of filler in the matrix as interfacial heterogeneity at the microscale, along with local mixing.

The connectivity index C was evaluated by identifying groups of adjacent filler pixels [10]. The area of each group was quantified as the number of connected pixels, and the connectivity index was defined as

(3) $$C=\frac{S_{max}}{S_{tot}}$$

where S_max_ is the area of the largest connected cluster, and S_tot_ is the total area of all clusters. The parameter C reflects the degree of network formation within the composite, with low values indicating isolated domains, and values approaching unity corresponding to a fully percolating filler network.

The combined use of global dispersion (D), connectivity (C), and local mixing (R) provides a comprehensive multi-scale description of the composite microstructure.

### 2.3. Raman Spectral Imaging

The Raman spectra were acquired via a micro-Raman confocal spectrometer (Horiba XploRA Plus, Horiba Scientific, Longjumeau, France). To avoid heat influenced alteration of the Raman spectra and damage to the samples, all the Raman spectra were excited with a 532 nm laser and a nominal laser power of 1.5 mW in the range 100–3500 cm^−1^. To obtain the detailed structural properties, the samples were scanned point by point in a prefixed grid, selecting areas down to 20 × 20 µm^2^ using an objective with 50× magnification and up to 700 × 700 µm^2^ using an objective with 10× magnification. The Raman spectra were processed via smoothing and polynomial background substraction using Labspec 6.7.2 software. The Raman spectrum of GO is typically characterized by two prominent bands: the D band, located around 1360 cm^−1^, and the G band, at approximately 1580 cm^−1^ [11,12,13,14]. The G band originates from the in-plane vibration (E_2_g mode) of sp^2^-hybridized carbon atoms, whereas the D band is associated with defects, structural disorder, and the presence of oxygen-containing functional groups [15,16]. In GO, the hydroxyl, epoxy, carbonyl, and carboxyl groups disrupt the extended sp^2^ conjugated network of the graphene structure and generate a high number of defects, leading to a relatively intense D band and an increased intensity ratio of the D and G band when compared to the results for pristine graphite. In contrast, the 2D band in GO, typically observed around 2650–2750 cm^−1^ in graphene and graphite, is often weak, broadened, or completely absent [17,18]. The 2D band is a second-order Raman process that requires a well-preserved and extended sp^2^ electronic structure to satisfy the double-resonance scattering mechanism. In GO, the extensive oxidation and fragmentation of the aromatic domains severely disrupt the electronic band structure, reducing the coherence length of the sp^2^ regions and suppressing the double-resonance process. Consequently, the 2D band either disappears or becomes extremely broad and of low intensity. The absence of a well-defined 2D band is therefore generally considered evidence of the highly defective nature of GO and confirms the substantial oxidation of the parent graphitic structure [15,16,17,18]. The intensity ratio I(D)/I(G), the G peak position, and other spectral features can be calculated for each spectrum trace. The Labspec program automatically provides the value distribution from which average value and quadratic dispersion are calculated for each map [19].

### 2.4. Monte Carlo and XCOM Modeling of Radiation Attenuation in PEI/GO Composite

The evaluation of the X-ray shielding and transmission performance of the PEI/GO composites was conducted using a dual computational approach, i.e., a deterministic model based on the classic photon attenuation formulation and a stochastic Monte Carlo simulation code mimicking experimental conditions. The geometry was configured to replicate an experimental transmission setup in which X-ray photons are generated from a virtual point source directed orthogonally toward the sample face under narrow-beam conditions.

The theoretical X-ray transmission (*T_theo_*) through the composite target was modeled as a function of incident photon energy (*E* = 10, 15, 20, 30, 40, 50 and 60 keV) using the Beer–Lambert law:

(4) $$T_{theo}=\frac{I}{I_{0}}=e^{-\mu_{comp}x}$$

where I and I_0_ represent the incident and transmitted photon intensities, respectively, and x denotes the physical sample thickness. The total linear attenuation coefficient of the composite, $\mu_{comp}$, was derived from the mass attenuation coefficients of the constituent elements according to the mixture rule:

(5) $$\mu_{comp}=\;\rho_{comp\;}\left[w_{PEI}{\left(\frac{\mu }{\rho }\right)}_{PEI}+\;w_{GO}{\left(\frac{\mu }{\rho }\right)}_{GO}\right]$$

where w*_i_* represents the mass fraction of each component, and the elemental mass attenuation coefficients ${\left(\frac{\mu }{\rho }\right)}_{i}$ were extracted from the NIST XCOM photon cross-sections database [20,21].

The composite target was modeled as a homogenous bulk plate, with an optimized density $\rho_{comp\;}$ calculated via the ideal rule of mixtures, using a PEI density $\rho_{PEI}$ = 1.03 g/cm^3^ and a structural volumetric density of the GO flakes, evaluated at $\rho_{PGO}$ = 1.31 g/cm^3^:

(6) $$\rho_{comp\;}={\left(\frac{w_{PEI}}{\rho_{PEI}}+\frac{w_{GO}}{\rho_{PGO}}\right)}^{-1}$$

As an example, for a 50 wt% GO content, $w_{PEI}\;=\;w_{PEI}\;=\;0.5,$ and $\rho_{comp}=1.15\;g/{\mathrm{cm}}^{3}$.

Monte Carlo radiation transport simulations model the stochastic nature of photon–matter interactions by explicitly tracking individual photon histories through a material. Each photon undergoes a sequence of free flights and interaction events sampled probabilistically from known interaction cross sections [22,23]. The computational parameters are reported in Table 1.

The total linear attenuation coefficient μ, which represents the fraction of radiation that is absorbed or scattered per unit thickness of the material [24], is decomposed into partial contributions associated with different physical mechanisms:

(7) $$\mu \left(E\right)\;=\;\mu_{phe}+\;\mu_{C}$$

where $\mu_{phe}$ and $\mu_{C}$ corresponds to photoelectric absorption and Compton scattering, respectively.

For each photon, the distance s to the next interaction is sampled from an exponential probability distribution:

(8) $$s=-\frac{ln(\xi )}{\mu_{comp}}$$

where ξ ∈ [0, 1] is a uniformly distributed random number. For each simulated history, the spatial trajectory is evaluated as follows:

(a) Transmission event—If s exceeds the sample thickness, the primary photon is recorded as successfully transmitted, contributing to the net transmission count *N_trans_*.
(b) Interaction event—If s is smaller than the sample thickness, the photon undergoes a deterministic interaction within the bulk material. In this regime, the branching ratio between absorption (photoelectric effect) and scattering (incoherent Compton and coherent Rayleigh phenomena) is evaluated using the respective probability cross sections extracted from the XCOM framework [21].

It is worth noting that photoelectric events lead to complete photon absorption and terminate the photon history, whereas Compton scattering events result in partial energy loss and angular deflection. The scattering angle is sampled from an approximate Klein–Nishina angular distribution. Photons scattered within a small angular cone relative to the incident direction (here < 30°) are classified as forward-scattered. These photons may still exit the material and contribute to the detected transmission, although with reduced energy.

This forward-scattering contribution explains why Monte Carlo simulations predict a slightly higher transmission than do the analytical Beer–Lambert models, which neglect angular redistribution effects.

### 2.5. Measurements of PEI/GO Composite X-Ray Attenuation

The X-ray irradiation of PEI/GO composites was performed at INFN-LNF XLab using an X-ray tube with a tungsten anode and a microfocus spot (5 μm) [25]. The X-ray projection images were acquired by a CareView 750 MT matrix detector by CareRay Digital Medical Technology (Suzhou, China) with a pixel size of 77 μm, an active matrix of 3072 × 4096 pixels, and a total area of 23.6 × 31.5 cm^2^. The beam configuaration was a cone-beam/broad-beam geometry with normal incidence on the flat specimen surface. The investigated samples were located at a 170 mm distance from the source, while the distance source to detector was 350 mm. It should be noted that the X-ray tube operates polychromatically, where the setting in kV represents the peak electron accelerating potential. The discrete photon energy values (E = 10–60 keV) used in the NIST XCOM and Monte Carlo calculations serve as monoenergetic baselines to evaluate the effective attenuation performance corresponding to the nominal operating voltages.

**Figure 2:**
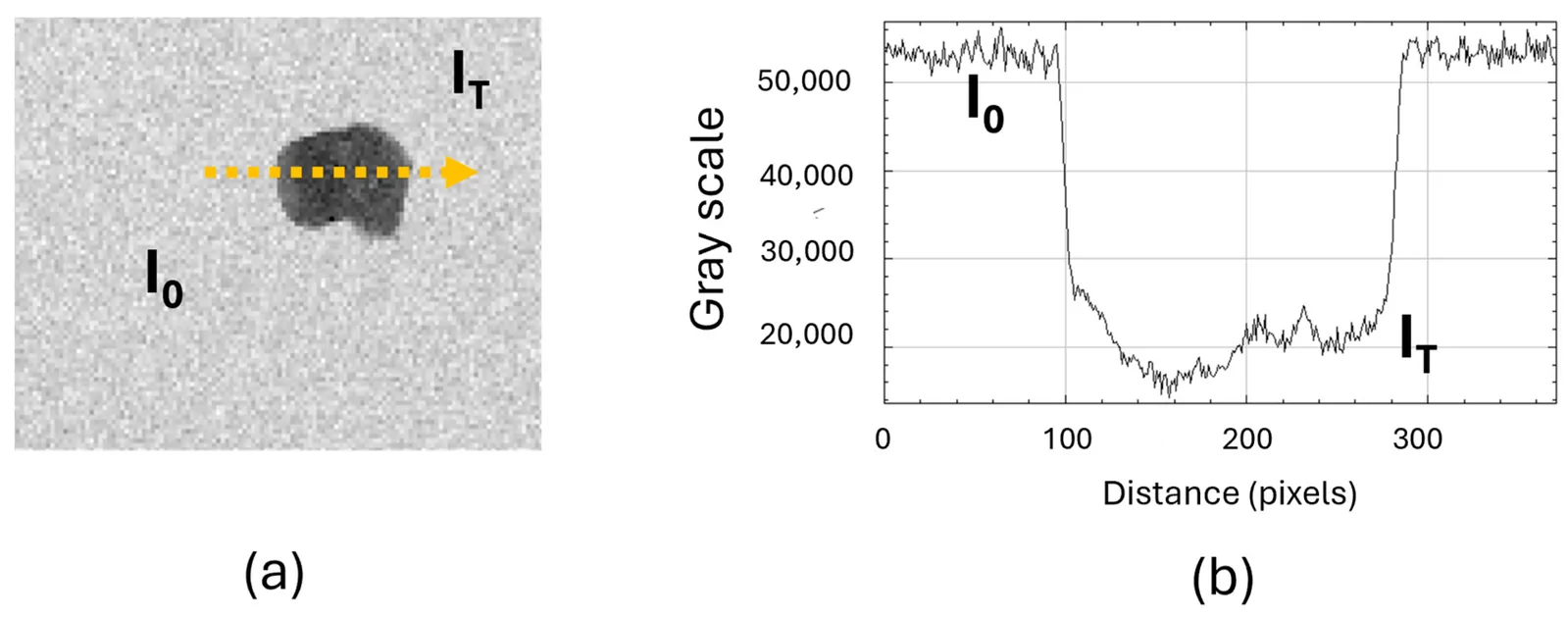
(**a**) Projection images composed by the transmitted radiation (I_T_) passing thorough the PEI/GO sample (GO = 50 wt%, thickness 0.9 mm) and the reference radiation (I_0_) propagating in the free space; (**b**) graph of the intensity profile of the image in (**a**), obtained along the dashed yellow arrow.

To evaluate the X-ray attenuation of the PEI/GO samples, a series of images was acquired at X-ray tube operating voltages ranging from 10 to 60 kV (10, 15, 20, 30, 40, 50 and 60), with the tube current maintained at 50 µA. Each image was acquired with an exposure time of 4 s (see Figure 2).

The projection images, comprising both the transmitted radiation (*I*_T_) passing through the samples and the reference intensity (*I*_0_) propagating in free space around the samples, were acquired as illustrated in Figure 2a.

The images were analyzed using Image J 1.53t (Wayne Rasband and contributors, National Institute of Health, Bethesda, MD, USA, http://imagej.nih.gov//ij, (accessed on 31 August 2026)) to extract the experimental transmission percentages at the specified photon energies. Figure 2b displays the intensity profiles extracted along the dashed yellow arrows indicated in Figure 2a.

## 3. Results

### 3.1. Microstructural Quantification and Raman Correlation

**Figure 3:**
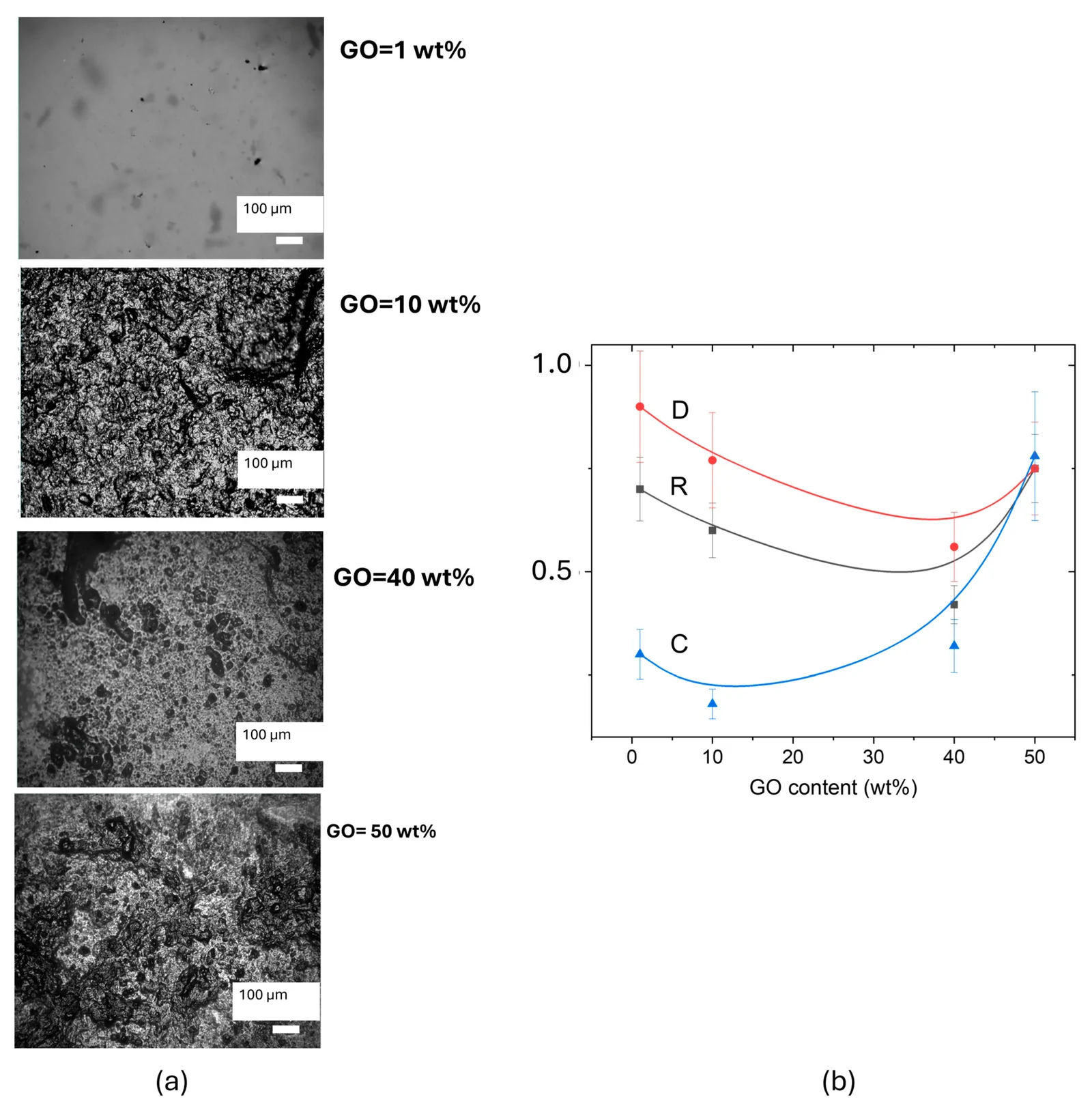
(**a**) Optical microscope images of PEI/composite samples with GO content ranging from 1 to 50 wt%; (**b**) structural parameters inferred by optical images reported in (**a**): dispersion index D calculated from binarized images, connectivity index C, and local mixing index R calculated from grayscale images.

Figure 3a reports the white light optical microscope images of PEI/GO composites with different GO content. From these maps, the structural parameters D, C, and R were extracted and reported in the plot of Figure 3b. At low GO contents, the PEI/GO sample exhibited the highest values for the D and R index, indicating a highly homogeneous distribution of isolated GO domains within the polymer matrix.

The connectivity index C appears to be unexpectedly high with respect to the other values for such a low filler content. This behavior can be attributed to thresholding effects during image segmentation, where intermediate grayscale regions are classified as filler, artificially enhancing the apparent connectivity. This result highlights an important limitation of connectivity metrics based solely on binary image analysis, emphasizing the need to interpret this parameter in conjunction with the dispersion index, particularly in low-concentration systems. At intermediate loading, the dispersion D and local mixing index R decreased, indicating the onset of local aggregation and the formation of extended GO-rich regions. At higher loading (40 wt%), the microstructure undergoes a transition toward a significant aggregation of GO disconnected clusters, as evidenced by the lowest dispersion index and the increase in connectivity value. By further increasing the GO content, the indexes D and R slightly increase, while the connectivity index C reaches the highest value, suggesting extensive coalescence in large, interconnected GO domains and partial percolation within the matrix. The observed behavior is consistent with that in previous studies indicating that increasing graphene-based filler content promotes restacking and aggregation due to strong van der Waals and π–π interactions [2,26].

**Figure 4:**
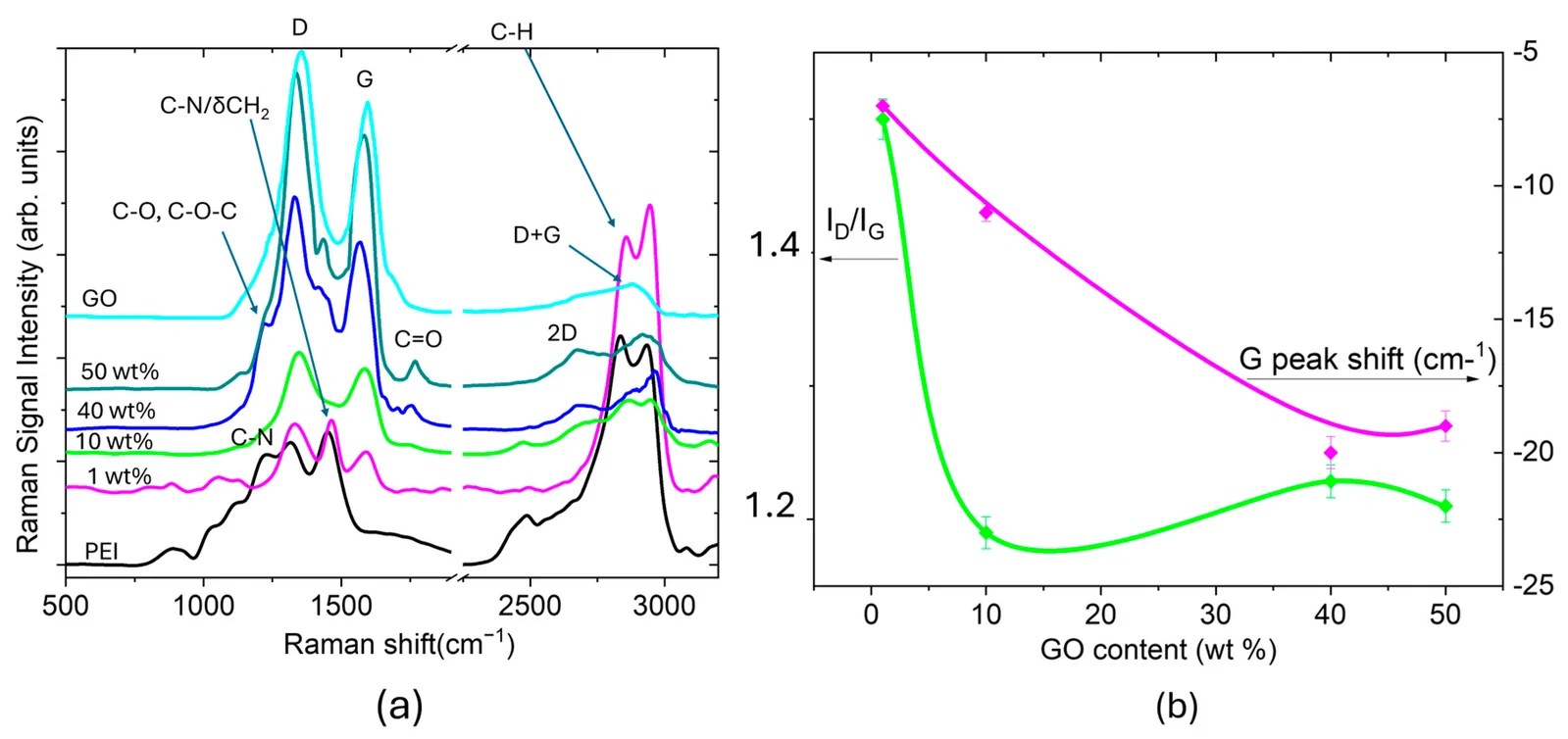
(**a**) Raman spectra of PEI, GO flakes, and PEI/GO composite samples with GO content ranging from 1 to 50 wt%. The Raman traces of the PEI and 1 wt% GO sample are amplified by a factor of 2 and 5, respectively, for better evidencing the first part of the spectra. (**b**) Values of the I_D_/I_G_ ratio and G peak shift (with respect to the position in the pristine GO flake) as a function of GO content in the composites.

Figure 4a reports the Raman spectra of the PEI, GO and PEI/GO composites. The pristine PEI spectrum is characterized by broad bands in the 1000–1500 cm^−1^ and 2800–3000 cm^−1^ regions, associated with C–N stretching, CH_2_ bending, and aliphatic CH vibrations, while the GO spectrum exhibits the typical D (~1350 cm^−1^) and G (~1580 cm^−1^) bands, associated with disordered and graphitic sp^2^ carbon domains [17,18,27,28]. Upon GO incorporation, these bands gradually emerge in the PEI/GO Raman trace and evolve depending on the filler concentration.

At 1 wt% GO content, the D and G bands are weak, although clearly detectable, indicating that the sp^2^ domains are limited in size and mostly isolated within the polymer matrix. The Raman spectrum, dominated by polymer vibrations, does not exhibit peaks that are not ascribable or to PEI or to GO, indicating that the GO sheets mainly interact with the PEI matrix via hydrogen bonding (N–H···O), with poor connectivity. As shown in Figure 4b, the ratio between the D and G band intensity is larger than the value of 1.2 measured in the pristine GO flake, while the G peak position is redshifted due to the charge transfer induced by the chemical functionalization. By increasing the GO content up to 10 wt%, a marked increase in both D and G band intensities is observed, together with improved definition of their spectral profiles. The I_D_/I_G_ ratio decreases, while the G peak shift increases. The interfacial reactions start at a GO content of 40 wt%: in the composite Raman spectra, new bands appear at 1050–1220 cm^−1^ (C-O, C-O-C stretching) and 1740 cm^−1^ (C=O stretching) due to the formation of NH^3+−^-COO^−^ ionic bonds and epoxide ring opening reactions, increasing the D-band intensity, while simultaneous formation of larger sp^2^ domains enhances the G band. The reactions between PEI amines and GO carbonyl/epoxy groups further increase the redshift G band peak, as widely reported for amine-functionalized GO systems [29]. The concomitant loss of structural homogeneity is evidenced by an increase in the disorder D band, and the 2D band appears, indicating a reduction in GO. By further increasing the GO percentage in the PEI matrix, the 2D band increases, while the spectrum approaches that of pure GO, with intense D and G bands. The emergence of the 2D band indicates a partial restoration of the sp^2^ conjugated network, resulting from a synergistic reduction mechanism. This process is primarily driven by the nucleophilic attack of the abundant primary and secondary amine groups of PEI on the epoxide and hydroxyl groups of GO, kinetically thermally assisted during the 90 °C curing step. Moreover, the increasing C index points to the formation of large sp^2^ domains through extensive π–π stacking. The I_D_/I_G_ ratio stabilizes at the value of pristine GO, indicating an interplay between defect generation through covalent functionalization and ordering within clustered sp^2^ domains. The reduced contribution of polymer-related peaks suggests a diminished role of interfacial bonding, with the structure transitioning from a polymer-dispersed system to a GO-dominated network.

Therefore, the observed microstructural transition is strongly governed by chemical interactions between the PEI matrix and the functionalized GO sheets. At low GO content, the nanoflakes achieve high spatial dispersion D and high microscale mixing R, remaining isolated within the PEI matrix. This structure is supported by the corresponding Raman signatures, which display only weak hydrogen bonding interactions. By increasing the GO content, the amine groups of PEI readily undergo nucleophilic ring-opening reactions with epoxide groups and form ionic linkages with carboxyl moieties on the GO basal planes and edges; these interfacial interactions regulate sheet dispersion and prevent immediate restacking at low loadings [30]. The emergence of new vibrational modes at 1050–1220 cm^−1^ (C-C stretching) and 1740 cm^−1^ (C=O stretching) is consistent with covalent interfacial grafting via nucleophilic epoxide ring-opening and ionic NH_3_^+−^-COO^−^ bonding between the PEI primary/secondary amines and the peripheral functionalities of the GO flakes [29,30].

However, at the highest loading regime (50 wt%), this covalent functionalization competes dynamically with the thermodynamic-driven self-assembly and restacking of unreacted graphene domains via interlayer π–π stacking. This mechanism leads to a fully connected, partially percolating 3D network, as reflected by the maximum value of the connectivity index C and the appearance of a sharp, structural 2D Raman band, indicating localized graphitic ordering, while the persistence of the D band suggests that structural defects remain present.

While high-resolution micro-Raman mapping provides conclusive localized evidence of structural ordering and partial sp^2^ network restoration, further dedicated analyses will be conducted in a follow-up study to fully map interfacial functional group dynamics.

### 3.2. Monte Carlo and XCOM Modeling of Radiation Attenuation in PEI/GO Composites

The radiation shielding performance of graphene-based materials is governed by the fundamental interaction mechanisms between X-ray photons and matter, which, in the 5–50 keV energy range, are dominated by the photoelectric effect, Compton scattering, and elastic and inelastic interactions with bound electrons [22]. Although graphene is composed of a low-atomic-number element (carbon, Z = 6), its unique electronic structure, exceptionally high electron density, and two-dimensional crystalline morphology led to attenuation behaviors that differ significantly from those of conventional carbon-based materials.

At low photon energies, typically below ~15 keV, photoelectric absorption represents the dominant interaction mechanism. In this process, the incident photon is absorbed by a bound electron, which is subsequently ejected from the atom. The cross section σ follows this relationship:

(9) $$\sigma \propto Z^{n}\cdot E^{-3}\;\mathrm{where}\;n\approx 4–5$$

where Z is the atomic number, and E is the energy of the incident photon, as established by the photon interaction cross-section data compiled by Hubbell et al. and implemented in the NIST XCOM database [23,24]. In the photoelectric regime, the strong Z-dependence intrinsically limits the shielding efficiency of low-Z materials such as carbon. Nevertheless, graphene partially compensates for the low atomic number through its exceptionally high electron density per unit mass and the presence of an extended delocalized π-electron system. In sp^2^-bonded graphene, π electrons form a continuous electronic cloud over the basal plane, increasing the probability of photon–electron interactions compared to the likelihood of the occurrence of disordered or amorphous carbon. As a result, the probability of effective photoelectric interaction per unit mass in graphene-based systems can exceed that of other carbonaceous fillers such as carbon black or conventional carbon fibers.

Moreover, in the case of GO, the presence of oxygen-containing functional groups further enhances low-energy attenuation. Oxygen atoms increase the effective atomic number of the composite and introduce additional bound electrons, leading to a measurable increase in photoelectric absorption below ~15 keV, as consistently predicted by XCOM-based calculations and confirmed by Monte Carlo simulations.

At intermediate photon energies, approximately from 15 to 50 keV, Compton scattering becomes the dominant attenuation mechanism. In Compton interactions, photons are scattered by weakly bound or quasi-free electrons, transferring part of their energy and momentum; the Compton scattering cross section depends primarily on the number of electrons per unit volume *N*_e_:

(10) $$\sigma \propto N_{e}$$

This dependence makes graphene-based materials particularly attractive in the Compton-dominated regime because the dense hexagonal lattice of graphene provides a high concentration of electrons within a minimal mass and volume, resulting in efficient scattering, even in low-Z systems.

Another distinguishing feature of graphene is the presence of delocalized π electrons that behave as a quasi-two-dimensional electron gas. The photons can interact not only with individual atomic electrons but also with collective electronic excitations associated with this π-electron system. These interactions enhance inelastic scattering processes and increase the overall interaction probability per unit mass. This mechanism is significantly weaker in other carbon-based materials, where structural disorder, sp^3^ bonding, or electron localization reduce the effective interaction volume.

Consequently, graphene provides a more efficient radiation–matter interaction platform than do conventional carbon fillers, particularly when dispersed within a host matrix due to the formation of percolating networks, as discussed in the previous paragraph, which further increase the probability of multiple scattering events within relatively small thicknesses, leading to the angular redistribution and energy degradation of photons, contributing to effective attenuation, even when complete absorption does not occur.

**Figure 5:**
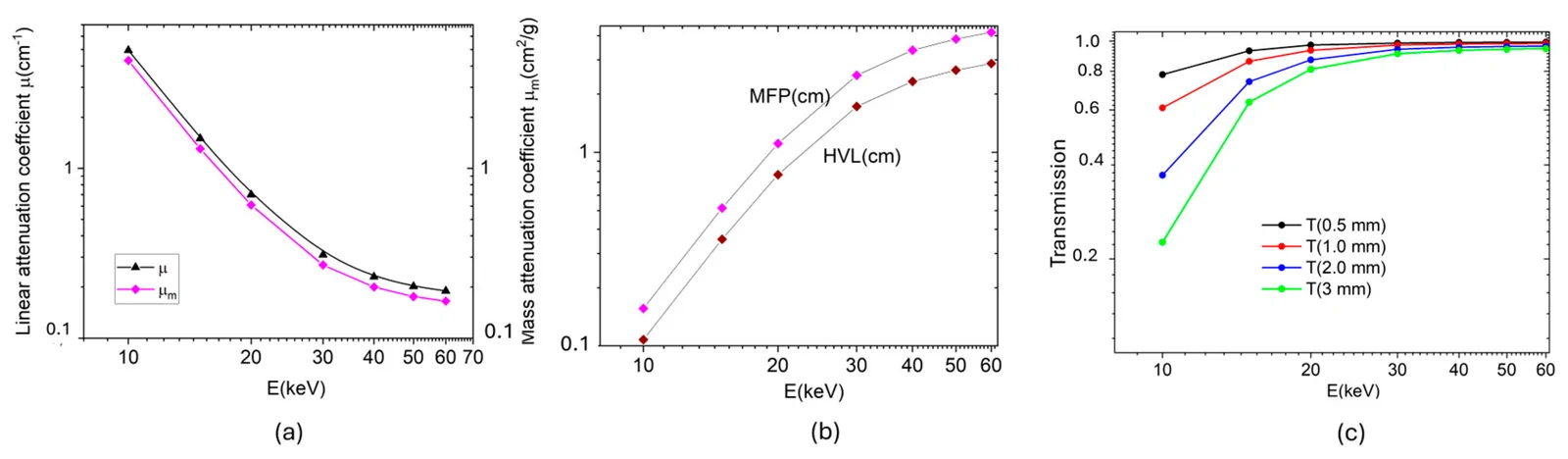
(**a**) Simulated linear µ and mass attenuation coefficient µ_m_ for photon transmission as a function of photon energy (10–60 keV) for the PEI/GO 50 wt% composite; (**b**) half value layer (HVL, brown diamonds) and mean free path values (fuchsia diamonds) derived from µ; (**c**) transmission curves obtained for different sample thicknesses: 0.5 mm (black circles), 1 mm (red circles), 2 mm (blue circles) and 3 mm (green circles). The transmission values include both uncollided and forward-scattered photons.

Figure 5a illustrates the simulated linear attenuation coefficient µ and the corresponding mass attenuation coefficient (μ/ρ) for PEI/GO 50 wt% composites over the 10–60 keV energy range. The half-value layer (HVL) and mean free path (MFP) were calculated according to the standard radiation shielding relationships:

(11) $$HVL=\frac{ln2}{\mu }$$

(12) $$MFP=\frac{1}{\mu }$$

and are reported in Figure 5b. The transmission values were also calculated for thicknesses ranging from 0.5 m to 3 mm (Figure 5c).

The mass attenuation coefficient µ_m_ follows the same trend as the linear attenuation coefficient because it is obtained by normalizing μ via the composite density; they provide identical information regarding the interaction physics, although the mass attenuation coefficient allows direct comparison with literature data, independent of sample density.

Both attenuation coefficients exhibit the characteristic monotonic decrease with increasing photon energy that is expected for low-Z polymer-based materials. At 10 keV, the composite exhibits the highest attenuation efficiency, with both μ and μ_m_ assuming their maximum values. This behavior reflects the predominance of the photoelectric effect, whose interaction probability is strongly dependent on photon energy and decreases approximately as *E*^−3^, although the exact dependence also depends on the elemental composition of the material. Consequently, even relatively small increases in photon energy produce a substantial reduction in the probability of photoelectric absorption. Between approximately 15 and 30 keV, both attenuation coefficients decrease rapidly, corresponding to the transition region in which photoelectric absorption progressively loses importance, while incoherent (Compton) scattering becomes increasingly significant. In this energy interval, the attenuation no longer depends exclusively on photon absorption but also on photon scattering, leading to a less pronounced energy dependence.

Above approximately 40 keV, both coefficients approach a nearly asymptotic behavior. In this region, Compton scattering becomes the dominant interaction mechanism within the composite, while the contribution of the photoelectric effect becomes comparatively small. Since the Compton cross section varies only slowly with photon energy, the attenuation coefficients exhibit a much more gradual decrease, resulting in the observed plateau-like behavior between 40 and 60 keV.

The calculated behavior is fully consistent with the theoretical interaction mechanisms reported in the NIST XCOM database and with previous studies on graphene-based polymer composites [30,31]. Similar energy dependence has been reported for GO/epoxy composites, graphene nanoplatelet/polymer systems, polyimide-based shielding materials and other lightweight carbon-filled composites, where attenuation is dominated by photoelectric absorption below approximately 30 keV and progressively controlled by Compton scattering at higher photon energies.

A monotonic increase in transmission with photon energy is observed for all samples, reflecting the decrease in the total attenuation coefficient as the relative contribution of the photoelectric effect diminishes, and Compton scattering progressively becomes the dominant interaction mechanism. The influence of thickness is particularly pronounced at low photon energy; at 10 keV, the transmission decreases from 0.73 to about 0.15 by increasing the thickness sample from 0.5 mm to 3 mm. This behavior is consistent with the exponential attenuation law and the high photoelectric interaction probability characteristic of low-energy photons. Under these conditions, even relatively small increases in thickness result in substantial reductions in transmitted intensity.

At higher energies (25–60 keV), all curves exhibit a tendency toward convergence. The transmission values increase progressively, and the differences between thicknesses become less pronounced. This behavior reflects the transition from photoelectric-dominated attenuation to Compton scattering, which is less sensitive to both material density and elemental composition.

Overall, the Monte Carlo calculations confirm that the PEI/GO 50 wt% composite behaves as a typical low-density carbon-rich shielding material, combining relatively high attenuation efficiency at low photon energies with the weight advantages required for aerospace and portable radiation-shielding applications. Although the theoretical attenuation coefficients exhibit the expected behavior predicted by photon interaction theory and are consistent with previous reports on graphene-based polymer composites, the comparison with the experimental attenuation coefficients presented in the following section reveals systematic positive deviations. This observation indicates that the intrinsic attenuation properties of the homogeneous composite cannot fully describe the behavior of the real heterogeneous material, emphasizing the important roles of GO organization, interfacial density, and photon transport geometry in determining the overall shielding performance.

### 3.3. Measurements of Radiation Attenuation in PEI/GO Composite with GO = 50 wt% Content

**Figure 6:**
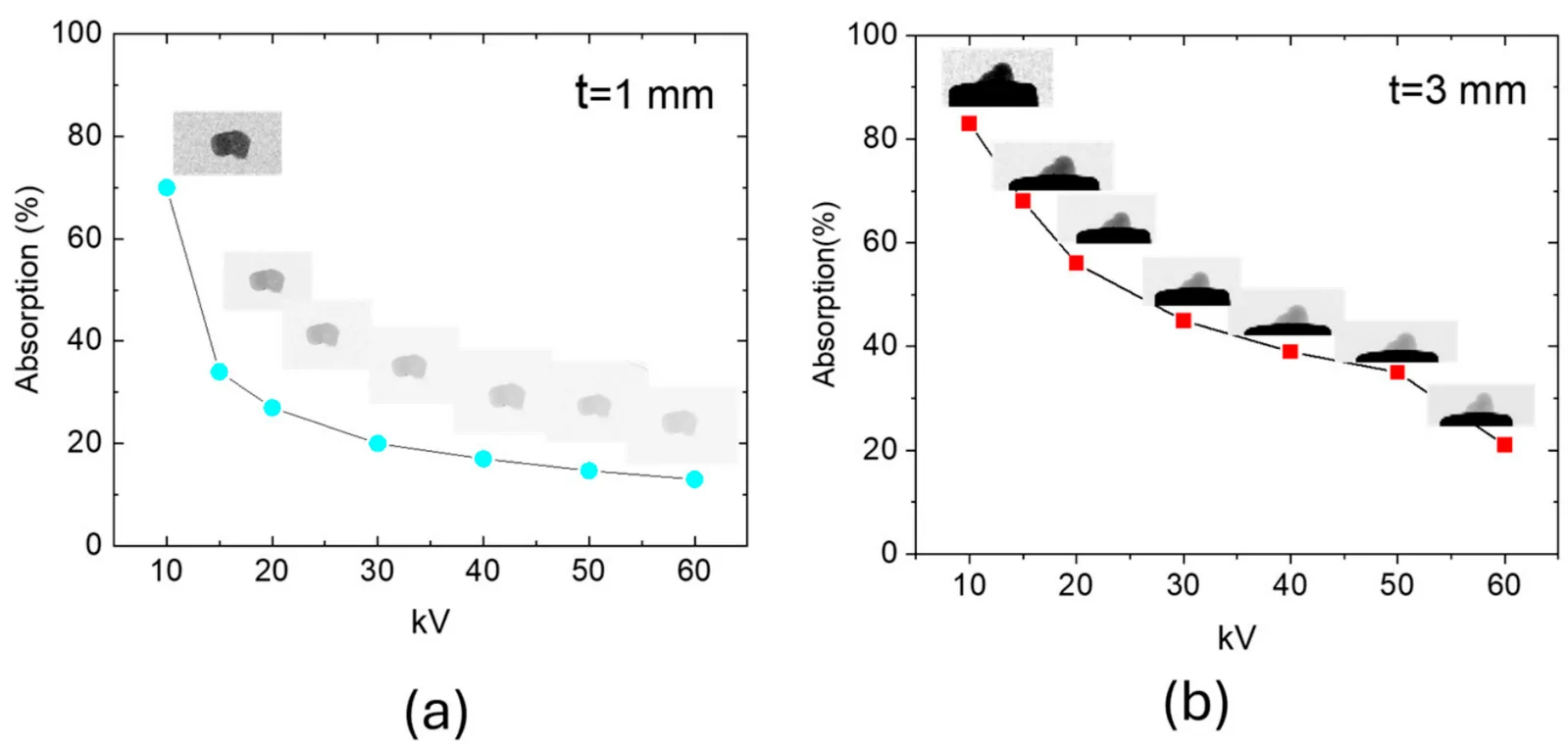
(**a**) Values of the absorption percentages for the PEI/GO composite with thicknesses of 1 mm and (**b**) 3 mm, respectively, at different X-ray tube operating voltages ranging from 10 to 60 kV.

Figure 6 reports the values of the absorption percentages for the samples of the PEI/GO composite with GO = 50 wt% with two different thicknesses. The samples were irradiated at different X-ray tube operating voltages ranging from 10 to 60 kV (10, 15, 20, 30, 40, 50 and 60). Insets show projection images of radiographic contrast on the detector.

**Figure 7:**
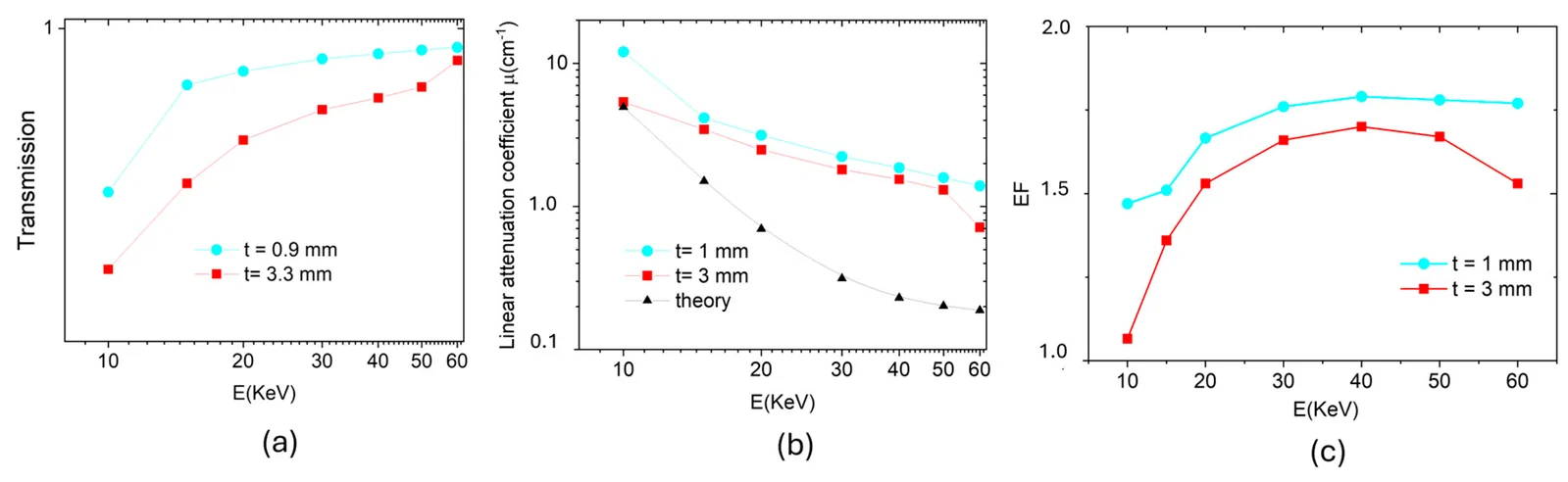
(**a**) Transmission values for PEI/GO composite samples with thicknesses of 1 mm (cyan circles) and 3 mm (red circles); (**b**) total linear attenuation coefficients calculated from transmission reported in (**a**), compared with the theoretical values (black triangles) at different photon energies. X-ray tube operating voltages ranging from 10 to 60 kV; (**c**) energy dependence of the attenuation enhancement factor (EF) for the PEI/GO (50 wt%) composite with thicknesses of 1 and 3 mm.

For a comparative discussion using literature and benchmark models, the corresponding transmission percentages were calculated and are reported as a function photon energy in Figure 7a. As expected, both configurations exhibit a monotonic increase in transmission with rising photon energy, which directly reflects the energy dependence of the total photon interaction cross section in low-Z polymeric matrices [22,23,24,31]. At lower energies (10–20 keV), the transmission drops sharply for both samples, reaching minimum values of approximately 0.30 and 0.17 for the 1 mm and 3 mm thick samples, respectively. This regime is heavily dominated by the photoelectric effect, where the cross section scales inversely with energy as E^−3.5^, resulting in a high mass attenuation coefficient [22,23,24]. As the energy shifts towards the medium-hard X-ray regime (30–60 keV), the photoelectric contribution rapidly decays, and incoherent (Compton) scattering becomes the primary interaction mechanism, causing the composite to become progressively transparent (T ~ 0.87 for 1 mm, and T ~ 0,78 for 3 mm at 60 keV). However, transmissions smaller than those predicted by the pure absorptive Monte Carlo model are observed. In this respect, it is worth noticing that the X-ray beam generated by the Xlab source is non-monochromatic; the presence of lower-energy spectral components below the nominal setting increases the effective interaction cross section of the incident radiation, thereby contributing to a further reduction in the measured transmission.

To draw a direct physical comparison, the experimental transmission data T for the 1 mm and 3 mm thick PEI/GO samples were converted into experimental linear attenuation coefficients using the relation:

(13) $$\mu =\;-\frac{\ln T}{t}$$

where *t* is the sample thickness, and *T* the transmission. These values are reported in Figure 7b through the theoretical curve. To account for potential local thickness variations and surface roughness in the high-filler-content specimens (50 wt%), thickness measurements were performed locally at the exact coordinate illuminated by the X-ray beam spot (5 μm). Local path lengths were determined by combining digital micrometer gauge readings with optical profiles cross-referenced to the radiographic contrast maps (see Figure 2), ensuring precise evaluation of µ.

In a narrow photon beam geometry passing through a perfectly homogeneous medium, the linear attenuation coefficient µ represents an intrinsic material property, meaning it should remain invariant, regardless of the sample’s physical thickness, while a distinct decoupling is observed between the µ values obtained for the 1 mm and 3 mm thick sample curves [22,23,24]. Moreover, an apparent physical paradox is observed: the thinner specimen (t = 1 mm, cyan curve) systematically displays a higher total linear attenuation coefficient than that of the thicker sample (t = 3 mm, red curve), implying that the thinner material absorbs “more efficiently” per unit length. This counterintuitive finding indicates the limits of ideal deterministic models when applied to X-ray propagation in real graphene composites [31,32].

To quantitatively evaluate the deviation of the experimental attenuation from the theoretical prediction, an attenuation enhancement factor (EF) was introduced as the ratio between the experimentally determined and theoretically predicted linear attenuation coefficients:

(14) $$EF=\frac{\mu_{exp}}{\mu_{theo}}$$

where EF = 1 corresponds to perfect agreement with the homogeneous Beer–Lambert model, whereas EF > 1 indicates enhanced attenuation in the real composite.

Figure 7c shows that the enhancement factor exhibits a well-defined dependence on photon energy for both investigated specimen thicknesses. Rather than fluctuating randomly around unity, EF increases rapidly between 10 and approximately 20–30 keV before approaching a broad plateau extending from approximately 30 to 60 keV. This systematic behavior strongly suggests that the observed deviation originates from intrinsic photon transport phenomena rather than from experimental uncertainty [32].

At low photon energies (10–20 keV), the attenuation process is dominated by photoelectric absorption, which is highly sensitive to local variations in elemental composition and electron density [22]. The heterogeneous distribution of GO domains within the PEI matrix therefore produces local fluctuations in the interaction probability that are not accounted for by the homogeneous mixture approximation adopted in the XCOM calculations [31,32].

Between 30 and 60 keV, where Compton scattering progressively becomes the dominant interaction mechanism, the enhancement factor reaches an approximately constant value of about 1.7–1.8 for the 1 mm specimen and about 1.6–1.7 for the 3 mm specimen. The existence of this plateau indicates that the additional attenuation mechanisms remain effective over the entire intermediate-energy range, suggesting that the contribution of the composite microstructure becomes nearly energy-independent once the transition from photoelectric absorption to incoherent scattering has been completed.

A second important feature is the systematic influence of specimen thickness. The thinner sample consistently exhibits larger enhancement factors than those of the thicker sample over the entire investigated energy range. This behavior indicates that the departure from the homogeneous attenuation model is more pronounced for short photon paths, whereas increasing thickness progressively averages the local density fluctuations and interface effects encountered by the photon beam [31,32]. Consequently, the effective attenuation approaches that predicted for an equivalent homogeneous medium as the propagation distance increases.

The nearly constant separation between the two curves above approximately 20 keV suggests that the enhancement factor is controlled not only by the intrinsic interaction cross sections but also by geometrical and microstructural effects, including the heterogeneous organization of GO, multiple polymer/GO interfaces, and broad-beam photon transport [31,32,33,34,35]. Although additional investigations employing phase-sensitive imaging or full Monte Carlo transport, including detector response, would be required to isolate the relative contribution of each mechanism, the present results clearly demonstrate that the shielding efficiency of graphene-based polymer composites cannot be fully described by conventional homogeneous attenuation models [31,33].

Overall, the enhancement factor provides a convenient quantitative parameter for evaluating the additional shielding effectiveness arising from the heterogeneous microstructure of graphene-based composites. Unlike the conventional attenuation coefficient, EF directly measures the deviation of the real material from the ideal homogeneous approximation and therefore represents a useful descriptor for comparing advanced multifunctional radiation-shielding materials [31,34].

The thickness dependence of the linear attenuation coefficient can be explained by the intrinsic nanoscale morphology of the PEI/GO composite in which, as observed in the previous paragraph, the clustering creates localized, high-density domains where percolative paths are separated by regions of pristine, lower-density PEI matrix [22,32]. The X-ray beam does not simply undergo standard photoelectric absorption in a homogeneous medium; instead, it is forced to cross multiple layers of extensive, interconnected percolative pathways of GO flakes along the beam propagation direction, experiencing refractions at the inner polymer-filler interfaces, maximizing the cumulative interaction probability, and causing an increase in the attenuation coefficient with respect to the calculated value.

In thicker samples, the effect of these interface-driven refractions is structurally attenuated by subsequent bulk layers, while in thinner samples, this effect is particularly important, shifting the cyan curve upward with respect to the red curve, particularly at smaller X-ray energy.

As the photon energy increases and Compton scattering takes over as the dominant event, the thicker sample triggers a pronounced Compton scattering buildup at higher energies (30–60 keV). In fact, the larger material volume promotes forward-directed, small-angle incoherent scattering events. The scattered photons are also captured by the wide field-of-view detector matrix, reducing the experimental attenuation coefficient of the 3 mm sample, pulling it down toward the ideal narrow-beam simulation line at 60 keV [23,33].

To further validate the radiation shielding efficacy of the synthesized PEI/GO composites, our experimental transmission curves were compared with the recent findings of Filak-Mędoń et al. [33], who investigated the total linear attenuation coefficients of an acrylonitrile butadiene styrene (ABS) matrix reinforced with 10 wt% graphene nanoplatelets (GNPs). Filak-Mędoń et al. reported a theoretical XCOM-derived linear attenuation coefficient of 0.218 cm^−1^ at 46 keV and experimental values declining to 0.151 cm^−1^ at 122 keV [33]. At intermediate energies close to the literature benchmark (46 keV), the 3 mm PEI/GO sample exhibits a linear attenuation coefficient of about 1.30 cm^−1^, which demonstrates a significantly enhanced attenuation capability compared to the 0.218 cm^−1^ value reported for the ABS/GNP system [32]. This prominent discrepancy is governed by two primary physical factors, i.e., the PEI matrix possesses a higher baseline density and a higher concentration of electronegative heteroatoms (such as oxygen and nitrogen) in its repeating imide units compared to the results for pristine ABS. Since the photoelectric cross section scales with the atomic number, following Equation (9), the PEI matrix yields a systematically higher attenuation capacity. Moreover, the GO flakes promote a different local interaction cross section compared to that of unfunctionalized GNPs [31].

The behavior observed by Filak-Mędoń et al., in which experimental measurements across different laboratories fluctuated based on detector geometries and geometric buildup factors, matches our observation that in the thin samples, forward-scattered Compton photons easily escape self-absorption within the matrix and reach the detector active area [32].

## 4. Discussion

When investigating the photon attenuation profiles, we observed that although the thicker sample (t = 3 mm) has a larger absorption, the thinner sample (t = 1 mm) systematically displays a higher total linear attenuation coefficient *µ* than that of the thicker bulk configuration, implying that the thinner material absorbs more efficiently per unit path length. Since the linear attenuation coefficient is an intrinsic material property, identical values would be expected for homogeneous materials under narrow-beam irradiation [22,23,24]. The observed thickness dependence therefore indicates that additional transport mechanisms contribute to the measured transmission.

A first contribution arises from the experimental acquisition geometry. While the Beer–Lambert formulation assumes an ideal narrow beam in which only uncollided photons reach the detector, the present measurements were performed under broad-beam conditions using a large-area flat-panel detector [25]. Under these conditions, forward-scattered photons generated through Compton interactions remain inside the detector acceptance angle and contribute to the transmitted intensity. This buildup effect becomes progressively more important as the sample thickness increases and naturally reduces the apparent attenuation coefficient extracted from experimental transmission measurements [23,33].

A second contribution originates from the heterogeneous microstructure of the PEI/GO composite. Optical image analysis and Raman spectroscopy demonstrate that GO does not form a perfectly homogeneous medium but rather evolves toward partially interconnected clusters separated by polymer-rich regions [31,32]. Consequently, the photon beam propagates through a medium characterized by continuous fluctuations of electron density and local composition rather than through the homogeneous material assumed by deterministic attenuation models [24,31]. Another effect may arise from interface-related wave propagation effects. The large number of PEI/GO interfaces generated by clustered GO domains can locally perturb the X-ray wavefront through refraction and edge-enhancement phenomena that have previously been reported in partially coherent X-ray imaging systems. When subjected to the coherent, paraxial wavefront of the microfocus source, these sharp phase boundaries maximize in-line refraction. The resulting wavefront interference diverts the primary photon flux away from the direct pixel collection path over the free-space propagation distance. In thin samples, where these interface-driven refractions are not structurally attenuated by subsequent bulk layers, this spatial intensity redistribution mimics an enhanced macroscopic absorption, shifting the thin-film attenuation curve upward. Although the present experimental configuration was not specifically designed to quantify phase-contrast effects, such mechanisms remain physically consistent with the enhanced attenuation observed in thin specimens, where interface-induced beam deviations are less likely to be averaged over long propagation paths. Conversely, as the physical thickness scales up, the 3D interconnected network establishes a more homogeneous radiological continuum—thereby smoothing out low-energy phase-contrast boundaries—concurrently triggering a pronounced Compton scattering buildup at higher energies [23,32]. The larger material volume heavily promotes forward-directed, small-angle incoherent scattering. This geometric buildup factor artificially inflates the recorded transmission, causing an underestimation of the intrinsic linear attenuation coefficient for the 3 mm sample.

Therefore, the apparent increase in the attenuation coefficient observed for thin samples should not be interpreted as evidence of intrinsically superior shielding efficiency but rather as the combined consequence of microstructural heterogeneity, broad-beam photon buildup and possible interface-induced wavefront perturbations [23,31,33].

## 5. Conclusions

This work successfully demonstrated the design, microstructural optimization, and radiation transport mechanisms of PEI/GO nanocomposites targeted for X-ray protection. By combining global binarization metrics with grayscale contrast algorithms and Raman spectral mapping, we tracked the transition from isolated, well-dispersed GO clusters to highly interconnected, percolating networks at filler concentrations exceeding 40 wt%. Confocal micro-Raman imaging confirmed that this structural evolution is actively driven by a competitive balance between covalent amine-functionalization (epoxide ring opening and carboxyl ionic pairing) and the graphitic restacking of large sp^2^ domains via π–π interactions. The formation of this network governs the transport physics of incident X-ray photons. X-ray attenuation measurements, paired with Monte Carlo and XCOM modeling, verified that high-content GO composites serve as effective, lead-free absorber-scatterers in the investigated radiation regime. The incorporation of GO drastically enhances photoelectric absorption below 20 keV, enabling the 3 mm sample with 50 wt% GO to shield 70% of incoming flux at 15 keV. Furthermore, the investigation reveals that Compton scattering forward-buildup in thick specimens and in-line refraction at matrix—filler interfaces in thin specimens are responsible for the thin-film linear attenuation coefficient increase.

Finally, it is important to outline the scope and limitations of these findings for operational space applications. In actual deep-space environments, radiation fields are composed of high-energy cosmic rays, solar particles, protons, and heavy ions. While these lightweight, flexible PEI/GO architectures represent a highly viable and attractive platform for the development of smart shielding textiles because they are highly effective at absorbing secondary X-rays and bremsstrahlung photons generated when high-energy charged particles collide with metallic structures, they must be integrated into multi-layered shielding architectures (e.g., combined with high-Z or hydrogen-dense structural layers) to ensure comprehensive radiation protection for crew suits and habitat liners.

## Figures and Tables

**Table 1:** Computational parameters used in the Monte Carlo radiation transport simulation. Note: Data and stochastic code execution were implemented via an advanced AI-assisted computational framework cross-verified with Geant4 standard benchmarks.

Simulation Parameter	Value
Photon histories	10^6^ per energy
Photon energies	10, 15, 20, 30, 40, 50, 60 keV
Material density	1.15 g/cm^3^
Sample thicknesses	0.5–3 mm
Interaction cross sections	NIST XCOM database
Interaction mechanisms	Photoelectric absorption and Compton scattering
Geometry	Planar slab, normal incidence
Output quantity	Photon transmission and linear attenuation coefficient
forward acceptance cone	<30°
Statistical uncertainty	<0.3%

## Data Availability

The original contributions presented in this study are included in the article. Further inquiries can be directed to the corresponding author.
